# Investigation of monogenic causes of familial breast cancer: data from the BEACCON case-control study

**DOI:** 10.1038/s41523-021-00279-9

**Published:** 2021-06-11

**Authors:** Na Li, Belle W. X. Lim, Ella R. Thompson, Simone McInerny, Magnus Zethoven, Dane Cheasley, Simone M. Rowley, Michelle W. Wong-Brown, Lisa Devereux, Kylie L. Gorringe, Erica K. Sloan, Alison Trainer, Rodney J. Scott, Paul A. James, Ian G. Campbell

**Affiliations:** 1 Cancer Genetics Laboratory, Peter MacCallum Cancer Centre, Melbourne, VIC Australia; 2 Sir Peter MacCallum Department of Oncology, The University of Melbourne, Melbourne, VIC Australia; 3 Drug Delivery Biology, Monash Institute of Pharmaceutical Sciences, Monash University, Melbourne, VIC Australia; 4 Department of Pathology, Peter MacCallum Cancer Centre, Melbourne, VIC Australia; 5 Parkville Familial Cancer Centre, Peter MacCallum Cancer Centre and Royal Melbourne Hospital, Melbourne, VIC Australia; 6 Bioinformatics Core Facility, Peter MacCallum Cancer Centre, Melbourne, VIC Australia; 7 School of Biomedical Sciences and Pharmacy, University of Newcastle, Callaghan, NSW Australia; 8 Lifepool, Peter MacCallum Cancer Centre, Melbourne, VIC Australia; 9 Cancer Genomics Program, Peter MacCallum Cancer Centre, Melbourne, VIC Australia; 10 Division of Surgery, Peter MacCallum Cancer Centre, Melbourne, VIC Australia; 11 Discipline of Medical Genetics and Centre for Information-Based Medicine, The University of Newcastle and Hunter Medical Research Institute, Newcastle, NSW Australia; 12 Division of Molecular Medicine, Pathology North, Newcastle, NSW Australia

**Keywords:** Breast cancer, Cancer genetics

## Abstract

Breast cancer (BC) has a significant heritable component but the genetic contribution remains unresolved in the majority of high-risk BC families. This study aims to investigate the monogenic causes underlying the familial aggregation of BC beyond *BRCA1* and *BRCA2*, including the identification of new predisposing genes. A total of 11,511 non-BRCA familial BC cases and population-matched cancer-free female controls in the BEACCON study were investigated in two sequencing phases: 1303 candidate genes in up to 3892 cases and controls, followed by validation of 145 shortlisted genes in an additional 7619 subjects. The coding regions and exon–intron boundaries of all candidate genes and 14 previously proposed BC genes were sequenced using custom designed sequencing panels. Pedigree and pathology data were analysed to identify genotype-specific associations. The contribution of *ATM*, *PALB2* and *CHEK2* to BC predisposition was confirmed, but not *RAD50* and *NBN*. An overall excess of loss-of-function (LoF) (OR 1.27, *p* = 9.05 × 10^−9^) and missense (OR 1.27, *p* = 3.96 × 10^−73^) variants was observed in the cases for the 145 candidate genes. Leading candidates harbored LoF variants with observed ORs of 2–4 and individually accounted for no more than 0.79% of the cases. New genes proposed by this study include *NTHL1*, *WRN*, *PARP2*, *CTH* and *CDK9*. The new candidate BC predisposition genes identified in BEACCON indicate that much of the remaining genetic causes of high-risk BC families are due to genes in which pathogenic variants are both very rare and convey only low to moderate risk.

## Introduction

The hereditary contribution to breast cancer (BC) is among the highest for solid tumours^1^. It is essential to identify the full repertoire of genetic risk factors to accurately inform BC risk management and interventions that can dramatically reduce risk^2^. However, two decades after the discovery of the *BRCA1/2*, only a small number of additional genes have been discovered and the genetic cause remains unresolved for the majority of hereditary BC families. Exome sequencing studies, focused on multi-case families, have revealed marked heterogeneity, which is the likely explanation for the lack of success of previous gene discovery studies where samples sizes and gene lists were generally small^3,4^. Consequently, we conducted the BEACCON study (hereditary BrEAst Case CONtrol study) to investigate all genes supported by either biological or empirical evidence. This resulted in a comprehensive targeted sequencing effort that examined 1303 candidate BC predisposition genes and 14 previously proposed hereditary breast and ovarian cancer (HBOC) genes in up to 5770 non-BRCA1/2 index cases and 5741 cancer-free population controls, providing a highly powered survey of the monogenic contributions to the heritable risk of BC.

## Results

### BEACCON study strategy

**Fig. 1:**
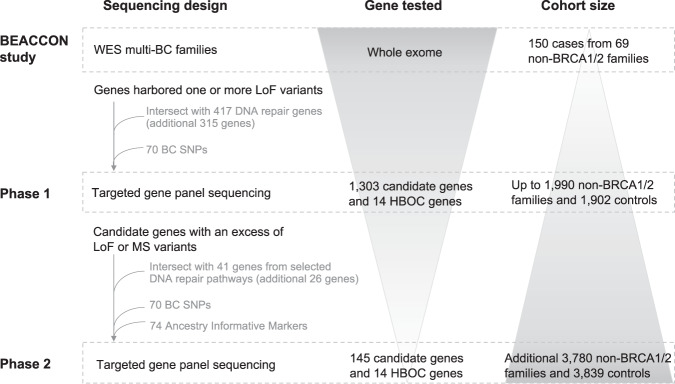
Breast cancer predisposition gene discovery and validation strategy in the BEACCON study. Whole-exome sequencing (WES) was carried out on 150 BC affected cases from 69 high-risk BC families. Based on the data from the WES and DNA repair genes identified through literature review, 1303 candidate genes and 14 previously reported HBOC genes were screened in up to 1990 index non-BRCA1/2 familial BC cases and 1902 controls. One hundred forty-five genes selected from phase 1 and the same 14 HBOC genes are screened in an independent cohort of 3780 index non-BRCA1/2 BC cases and 3839 controls.

The BEACCON study was conducted in two phases (Fig. 1). Phase 1 sequenced 14 previously reported HBOC genes (*CHEK2, PALB2, ATM, TP53, RAD51C, RAD51D, CDH1, BARD1, PTEN, MRE11A, BRIP1, STK11, RAD50* and *NBN*) and 1303 candidate genes (Supplementary Data) in up to 1990 non-BRCA1/2 familial BC index cases and 1902 female controls. The genes analysed consisted of 988 identified through previous BC germline exome studies^3,5^, and an additional 315 included because of a reported role in DNA damage repair^6–8^. Common variants are excluded (LoF MAF ≥ 0.005 and MS MAF ≥ 0.001) and the burden of remaining variants in candidate genes were compared between cases and controls to determine a short list for gene discovery purposes. A total of 145 genes from phase 1 were selected for further study based on the most statistically significant enrichment in cases (119 genes) or at a lower level of significance but with additional existing support based on current literature (26 genes). Together with the 14 HBOC genes these candidates proceeded to phase 2 and were sequenced in an additional 3780 non-BRCA1/2 cases and 3839 controls (159 genes analysed in a total of 5770 cases and 5741 controls). Subjects in both case and control cohorts were predominantly of European ancestry (95.3% cases and 98.8% controls) based on principal component analysis (PCA) (Supplementary Fig. 1).

An average sequencing depth of 257.5 and 10× sequencing coverage of 92.4% (cases 92.0%, controls 92.9%) was achieved, with no coverage bias between the cases and controls identified at an individual gene level or between sequencing phases. PLINK identity-by-state analysis was used to identify duplicated or closely related samples.

### Established moderate penetrance BC genes

**Fig. 2:**
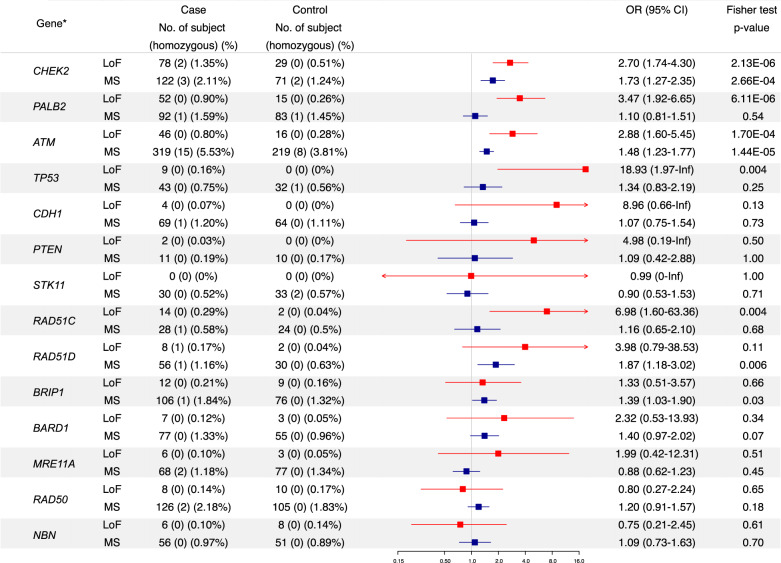
Case-control frequencies of rare LoF (MAF < 0.005) and MS (MAF < 0.001) variants in known or proposed HBOC genes (*N* = 14). *Part of the data have been published previously in *J. Clin. Oncol*. 34(13), 1455–1459, *Genet. Med*. 21(4), 913–922, *J. Pathol.* 245(1), 53–60, *J. Natl Cancer Inst*. 111(12), 1332–1338, *Nat. Genet*. 50(10), 1346–1348 and *Breast Cancer Res. Treat*. 159(2), 385–392. Known pathogenic MS variants in *ATM* (c.7271 T > G) and *TP53* (c.524 G > A, c.712 T > C, c.725 G > A, c.733 G > A, c.742 C > T and c.1009 C > T) were classified as LoF variants in this analysis.

Pathogenic LoF variants in *CHEK2*, *PALB2* and *ATM* were observed in 1.35%, 0.90% and 0.80% of cases with ORs of 2.70, 3.47 and 2.88 (Benjamini–Hochberg adjustment, BH *p* = 0.0003, 0.0005 and 0.009), respectively (Fig. 2), in agreement with published evidence^9,10^. Rare missense (MS) variants were found in significant excess in *CHEK2* (2.11%, 122 cases versus 1.24%, 71 controls; OR 1.73, 95% CI 1.27–2.35, BH *p* = 0.01) and *ATM* (5.53%, 319 cases versus 3.81%, 219 controls; OR 1.48, 95% CI 1.23–1.77, BH *p* = 0.0008). Applying filters for population frequency and in silico pathogenicity prediction scores to the rare MS variants, identified progressive enrichment of potentially deleterious variants in *CHEK2* and *ATM* in the cases (Supplementary Table 1). In contrast, *PALB2* showed neither an overall excess of MS variants in cases nor in any of the reported functional domains^11,12^.

### Cancer syndrome genes

*TP53, CDH1, PTEN* and *STK11* are rare high-risk genes that predispose to multi-cancer syndromes that include BC^13–15^. Pathogenic variants in *TP53* were detected in nine cases (0.16%), *CDH1* in four cases (0.07%) and *PTEN* in two cases (0.03%) but none were detected in controls for these genes and no *STK11* pathogenic variants were found in cases or controls. Given the rarity of mutations, the BC risk associated with these genes, although high, could not be accurately estimated and their collective contribution to the hereditary risk of BC is small.

### Proposed BC genes frequently tested on clinical multi-gene panels

There is less established evidence on the roles of *RAD51C, RAD51D, BRIP1, BARD1, MRE11A, RAD50* and *NBN* in BC predisposition^16–20^; however, these genes are included on many HBOC gene panels in clinical practice. In the BEACCON study *RAD51C* LoF variants were highly enriched in cases; 14 cases vs 2 controls (OR 6.98, 95% CI 1.60–63.36) (Fig. 2 and previously reported in ref. ^21^). A non-significant excess of LoF variants were detected in the cases for *RAD51D*, *BRIP1*, *BARD1* and *MRE11A*, and a statistically significant (unadjusted *p* < 0.05) excess of rare MS variants were identified in *RAD51D* and *BRIP1*. In contrast, there was no excess of variants in *RAD50* and *NBN* which form the MRN complex with *MRE11A*; the LoF variant frequency in *RAD50* and *NBN* was higher in the control group, indicating that, in the Australian population there is no evidence that these genes contribute to BC predisposition.

### Identification of candidate genes from the BEACCON data

Although many of the genes tested in phase 1 showed a higher frequency of LoF and MS variants in cases (Supplementary Fig. 2), none of the 1303 candidate genes or the 14 proposed HBOC genes passed the multiple testing corrected statistical significance level. However, the adjusted frequency of rare variants detected across all the candidate genes was significantly greater in the cases (LoF: OR 1.13, *p* = 7.42 × 10^−5^; rare MS: OR 1.17, *p* = 8.62 × 10^−55^). This enrichment was confirmed for the final 145 candidate genes in phase 2 alone (LoF OR 1.09, *p* = 0.05; MS OR 1.26, *p* = 3.83 × 10^−57^) and combined phase 1 and 2 data (LoF OR 1.27, *p* = 9.05 × 10^−9^; MS OR 1.27, *p* = 3.96 × 10^−73^) (Supplementary Table 2).

**Fig. 3:**
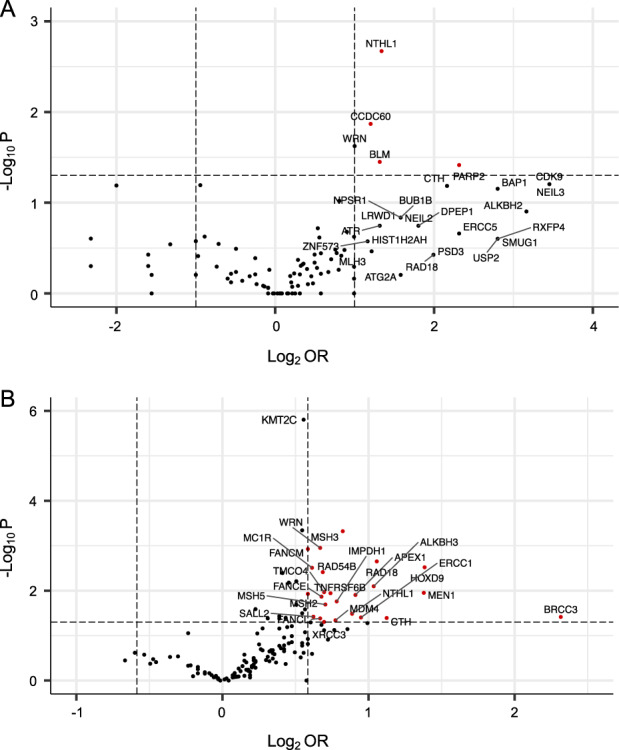
Distribution of candidate genes by ORs and *p*-values for LoF and MS variants. Volcano plots of the distribution of candidate genes (*N* = 145) by ORs and *p*-values in the LoF variant analysis (**A**) or the MS variant analysis (**B**). Genes are predominantly sequenced in a minimum of 4807 cases and 4782 controls with individual samples size listed in Supplementary Data. The horizontal axis is the log2(OR) and the vertical axis represents the reliability of the result (−log10(P)). The horizontal dash line signals Fisher’s exact text *P*-value at 0.05. Two vertical dash lines show the thresholds of ORs (OR = 2 and =0.50 for LoF variants and OR = 1.50 and =0.67 for MS variants). Each dot represents a candidate gene and the red dots represent the genes that have a minimum fold excess of variants in the cases and have passed the *p*-value threshold.

Five candidate genes (*NTHL1, CCDC60, WRN, BLM* and *PARP2*) showed an excess of LoF variants and 37 showed an excess of MS variants in the cases (unadjusted *p* < 0.05; Fig. 3, Supplementary Fig. 3). Two genes (*NTHL1* and *WRN*) were enriched for both LoF and MS variants. The 10 genes most enriched for MS variants (*KMT2C*, *MSH3, WNK1, WRN, FANCM, RAD18, HOXD9, MC1R, RAD54B* and *SLEX4)* remained significant upon multiple testing adjustment (Fig. 3, Supplementary Data). A high proportion of the candidate genes identified through an excess of LoF variants also exhibited an excess of MS variants, consistent with the findings for the HBOC genes. However, few of the candidate genes identified through an excess of MS variants had a corresponding excess of LoF variants (Supplementary Fig. 3).

**Fig. 4:**
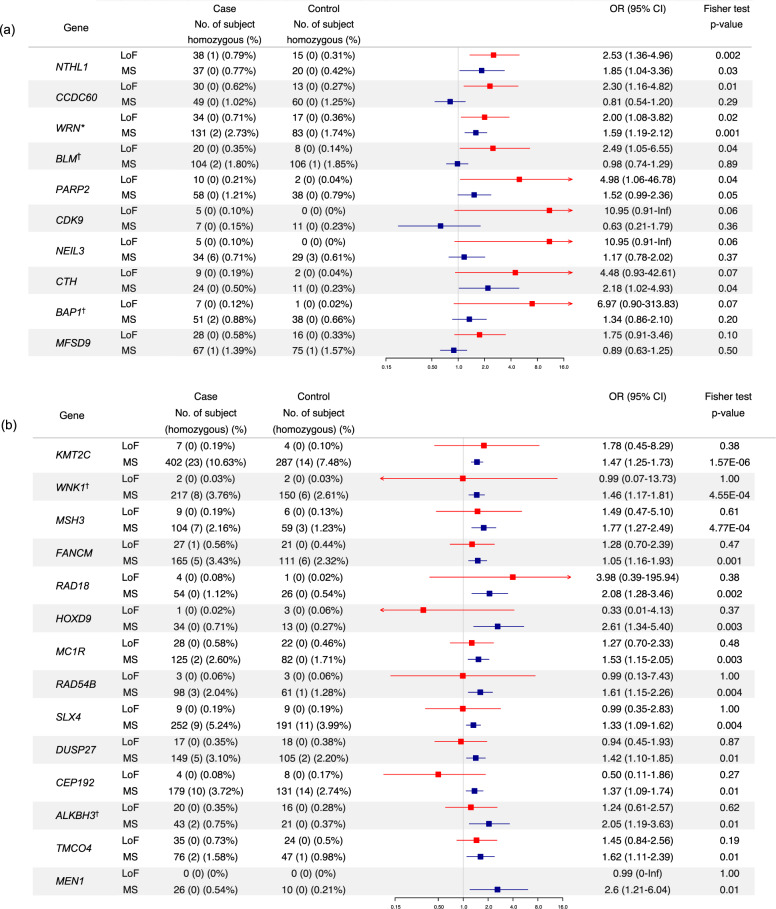
Top candidate genes ranked by *p*-value according to the excess of a rare LoF and MS variants. **a** (*p* ≤ 0.10, *N* = 10) and **b** (*p* ≤ 0.011, *N* = 14). *One LoF variant in the gene WRN (p.Arg1406Ter) located in the last exon was detected in 21 cases and 11 controls. Although this variant is reported in gnomAD at high frequency in South Asian (MAF 0.0171 for South Asians compared to 0.0015 for Europeans), the identified carriers from this study are all of European origin except for one South Asian. BLM, BAP1, WNK1 and ALKBH3 were sequenced in 5770 cases and 5741 controls, and KMT2C was sequenced in 3780 cases and 3839 controls. The remaining genes were sequenced in 4807 cases and 4782 controls.

The top candidate genes ranked by p-values according to the excess of rare LoF variants and rare MS variants are shown in Fig. 4a and b, respectively. Details of the variant frequency of all candidate genes are summarised in Supplementary Data. The number of individuals where any LoF variant was detected was low for all of the candidate genes: only 2.75% of cases harboured one or more LoF variants in a candidate gene that had a *p*-value < 0.05. The estimated ORs for the leading candidates identified through LoF variants are consistent with moderate BC risk, although the confidence intervals are wide, and none of the associations are statistically significant after multiple tests correction. The frequency of MS variants detected in each gene varied widely, from less than 1% for *HOXD9* and *MEN1*, to over 10% for *KMT2C* but the estimated odds ratios fell consistently within the range of low-moderate penetrance (1.33–2.61).

Among the top 10 genes ranked on the basis of LoF variant enrichment, seven are involved in DNA damage repair: *NTHL1* and *NEIL3*, which play important roles in base excision repair (BER), *BLM*, *WRN* and *BAP1* in homologous recombination repair (HRR), and *PARP2* and *CDK9* in double strand break response. Collectively, genes involved in HRR were enriched in cases (17 genes, after exclusion of reported HBOC genes) (OR 1.48, 95% CI 1.18–1.91, *p* = 0.001), as were genes involved in BER (*n* = 17) (OR 1.32, 95% CI 1.04–1.71, *p* = 0.02) (Supplementary Table 3).

### Identification of candidate genes from subgroup analysis

**Fig. 5:**
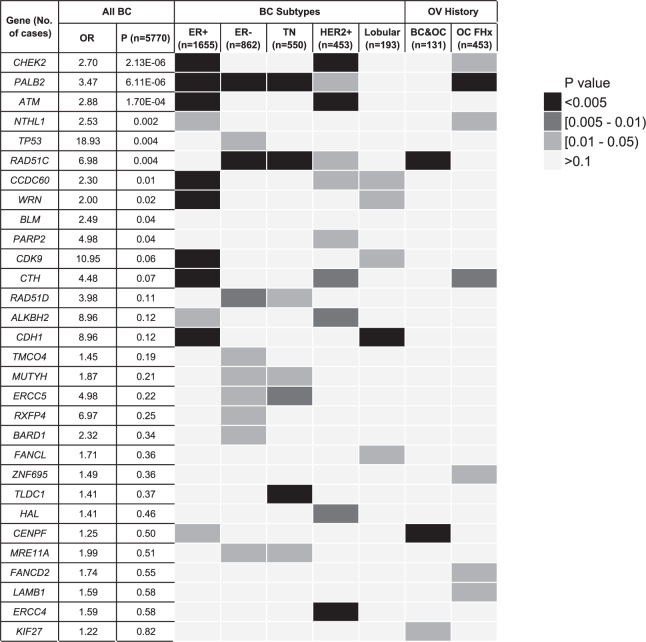
Heatmap of the associations of the candidate genes with specific subtypes of BC or a personal or family history of OC. Only LoF variants were considered in the analysis for candidate genes and HBOC genes (*N* = 159) in cases from ViP cohort and controls, and genes with one or more statistically significant associations (*p* < 0.05, Fisher’s exact test, two-sided) are listed.

Associations of the candidate genes with specific subtypes of BC or a personal or family history of OC were assessed for 3065 cases in the ViP cohort. Cohort characteristics including age at diagnosis, hormone receptor status and family history are summarised in Supplementary Table 4. The frequency of LoF variants in the 145 candidate genes and 14 HBOC genes were examined in five cancer subgroups (ER positive, ER negative, HER2 positive, triple negative (TN) and lobular BC), as well as personal or family history of OC, in comparison to the cancer-free controls (Fig. 5). Consistent with previous reports, *CHEK2* and *ATM* were correlated with ER positive^14,15,22,23^ and *CDH1* with lobular BC^24,25^. Associations with ER negative and TN BC were identified for *RAD51D*, *MUTYH, ERCC5*, *MRE11A* and *RAD51C*^21,26^. A number of genes such as *CHEK2*, *ATM* and *ERCC4* were correlated with HER2-positive BC. Genes associated with a personal or family history of OC included *RAD51C*, *PALB2* and candidate genes *CENPF, KIF27* and *CTH*.

### Carriers of multiple LoF variants

The study identified 188 (3.9%) cases and 111 (2.3%) of controls carrying LoF variants in multiple candidate genes (Supplementary Table 5A). The higher number of multiple LoF variant carriers in cases was not significantly different to the number expected based on the higher overall frequency of LoF variants in cases (*Χ*^2^ *p*-value = 0.52, Supplementary Table 5B) and the increased risk was consistent with a simple multiplicative effect: the OR increased from 1.31 to 3.16 as the number of variants carried by an individual increased from 1 to 3 (Supplementary Table 5A).

### Recurrent variants in candidate genes

Seventy-six percent of the LoF variants (*n* = 2564/3356) and 76% of the MS variants (*n* = 28,385/37,342) detected were unique in the cohort, and 83% of LoF variants and 78% of MS variants have a population frequency of <0.0001 in the GnomAD database. It is possible that certain recurrent variants might be driving the signal observed for some genes; however, the effect of the majority of individual variants was impossible to assess due to their rarity. We analysed 32 LoF and 136 MS variants that were detected more frequently (>0.1% in the overall BEACCON cohort and accounted for >10% in the variants in that gene), and residual gene odds ratios were calculated with the recurrent variants excluded (Supplementary Data). This analysis showed the known *CHEK2* pathogenic variant c.1100delC, with an odds ratio of 2.4, contributed 85% of all pathogenic *CHEK2*-carrying subjects. The residual *CHEK2* odds ratio was 6.98 (*p* = 0.004, without adjustment for multiple testing). No individual LoF variants were observed at a similar level as the *CHEK2* c.1100delC in any of the candidate genes. However a recurrent MS variants were identified in a number genes, including *APEX1, FANCE* and *RAD54B*. It should be noted that with the large number of MS variants analysed, these results would be consistent with chance findings and, with the exception of *APEX1* c.50 T > C, none remained significant upon multiple testing.

### Contribution to population breast cancer

To compare the relative contribution of different subgroups of coding variants to BC in the Australian population we estimated the population attributable fraction (PAF). Pathogenic variants in *BRCA1* and *BRCA2* were estimated (using published relative risks) to be responsible for 1.5% of BC, consistent with previous estimates^27,28^. Using the ORs and control frequency observed in this study, the combined contribution of pathogenic variants in *PALB2*, *CHEK2* and *ATM* was estimated to be similar (PAF 1.5%) while the contribution from high-risk syndromic genes was very small (PAF 0.2%), as was the remaining HBOC panel genes (*RAD51C*, *RAD51D*, *BARD1*, *BRIP1* and *MRE11A*, PAF for LoF variants 0.4%). In contrast, the excess of LoF variants in those candidate genes with at least a two-fold enrichment observed in cases versus controls (*n* = 26) corresponded to a PAF of 2.3%, while the collective effect of the excess of rare MS variants in phase 2 genes that showed at least a 1.5-fold enrichment summed to more than 12.3%. The same calculation applied to the published effect of polygenic risk score for BC^29^ in this group gives a PAF of 8.1%.

## Discussion

The BEACCON study aimed to address the lack of power of previous studies to identify additional BC predisposition genes by performing extensive sequencing in 12,000 women (11,511 analysed following exclusions) and further enhancing power by using an ‘extreme phenotype’ design with enrichment of familial non-*BRCA1/2* cases, compared with a control population of older women with ongoing confirmation of cancer-free status at June 2019. Three-quarters of the 1303 candidate genes screened were selected based on empirical evidence from local (69 multi-case BC families) or international whole-exome sequencing studies^3^, and the remainder were included to provide detailed coverage of functional pathways with established associations with BC.

While an overall enrichment in cases of LoF and MS variants was observed, this was distributed across many genes with no phase 2 candidate gene harboring LoF variants in more than 1% of the case cohort, with a median of only 0.15% (1 case in 667). The strongest candidate genes identified, including *NTHL1* and *WRN* were characterised by an excess of both LoF and MS variants in cases, and most were involved in some aspect of DNA repair or genomic stability, particularly the HRR and BER pathways consistent with the function of the established HBOC genes^30,31^. An interesting exception to this is the gene *CTH*, which has a role in the *trans*-sulfuration pathway where it regulates cellular oxidative stress^32^. Homozygous and compound heterozygous pathogenic MS variants in *CTH* are observed in the recessive metabolic disorder, cystathioninuria^33^, indicating that these variants are associated with reduction or loss of protein function. The finding of enrichment in cases for both rare MS and LoF variants in *CTH* is consistent with the possibility that reduced *CTH* activity may predispose to BC via perturbation of cellular oxidative stress leading to increased DNA damage^34^.

The large majority of genes that showed an excess of MS variants did not have a corresponding excess of LoF variants in the same gene, with the strongest candidates in this group being *KMT2C, WNK1, MSH3, FANCM* and *RAD18*^35,36^. Of these, *FANCM* has been a gene of interest in multiple studies^37–39^; however, these reports have focused on LoF variants that were not associated with breast cancer in this study (OR 1.28, 95% CI 0.7–2.39); although more than two thirds of the LoF variants observed in *FANCM* were two comparatively frequent distal nonsense variants (p.Gln1701Ter and p.Arg1931Ter). An excess of MS variants in the absence of enrichment for LoF variants may indicate a false-positive result, although the finding may also reflect a genuine predisposition effect due to other mechanisms, as seen with pathogenic variants in *TP53*.

Genetic predisposition to specific BC subtypes is increasingly recognized and may allow identification of predisposition effects that are undetectable in the analysis of all BC. This approach was validated by the detection of the established associations of *CHEK2* and *ATM* pathogenic variants with ER-positive tumours, and *CDH1* with lobular BC. For several candidate genes, potentially pathogenic variants were enriched in a specific phenotypic sub-cohort, despite not showing evidence of an association with BC in the overall case-control cohort, including the suggested associations for *ERCC5, RAD51D, MUTYH, MRE11A* and *TLDC1* with TN BC.

Limitations of the study may influence the interpretation of these results. An appreciation of population substructure and ethnicity is critical for studies of this nature. PCA demonstrated that BEACCON cases and controls were dominated by subjects of European origin and were directly comparable with the exception of a small difference in the Asian subgroup. In addition, while stringent quality filters were applied to select for variants of high confidence, it is possible some may still represent sequencing or alignment artefacts. A population frequency cut-off commonly used for gene discovery was implemented to prioritize a group of rare variants, which are more likely to represent moderate to high penetrance BC risk alleles (MAF ≤ 0.005 for LoF variants and MAF ≤ 0.001 for MS variants). These frequency cut-offs are arbitrary and some genuine pathogenic variants may escape this filtering and many variants below these cut-offs are likely to be non-pathogenic. Because the BEACCON study is enriched for familial cases, it is likely to overestimate the effect when compared to the general population and therefore it should be noted that the ORs detected do not equate to relative risks. Finally, the study did not investigate non-coding variants that affect gene regulation or splicing, large genomic rearrangement, or epigenetic changes that may also make important contributions to BC predisposition^40^.

In conclusion, the BEACCON study gives an insight into the scale required for future validation and discovery efforts that investigate rare coding variants. The low frequency of LoF and potentially pathogenic MS variants spread over a large number of different genes with apparently only moderate effect sizes meant that even with 11,511 subjects the evidence to support any candidate gene was limited. Applying the Benjamini–Hochberg adjustment for multiple comparisons, 10 candidate genes identified by an excess of MS variants, none of the candidate genes by LoF variants, and only *PALB2*, *CHEK2* and *ATM* among the HBOC genes, reached conventional statistical significance. Estimation of attributable risk indicated that a substantial component of the remaining heritable contribution to BC is found in coding variation, and particularly in large numbers of rare MS variants of minor effect that are difficult both to identify and to interpret. Cohort studies at least an order of magnitude greater in size will be required if case-control data alone are to resolve the remaining monogenic causes of BC predisposition. Additional lines of evidence, such as family segregation analysis and functional studies, will still be necessary to confirm a role in BC predisposition as has been demonstrated for *PALB2*, *ATM* and *RAD51C*^21,41,42^.

## Methods

### Study subjects and sequencing

Case subjects are female index patients diagnosed with BC and/or ovarian cancer from 5770 HBOC families ascertained by the Variants in Practice (ViP) Study from the combined Victorian and Tasmanian Familial Cancer Centres, Australia (*n* = 3065), or from the Pathology North, NSW Health Pathology, Newcastle, Australia (*n* = 2705). All cases were assessed by a specialist Familial Cancer Clinic and determined to be eligible for clinical genetic testing for HBOC genes (≥10% chance of a pathogenic variant), but tested negative for *BRCA1* and *BRCA2* pathogenic variants. Pathology reports and detailed pedigrees were analysed for the cases from the ViP cohort. Controls are 5741 cancer-free female subjects who were >40 years old from the Lifepool Study (http://www.lifepool.org/). The average age of diagnosis of the cases was 49.7 years (range 19.0–94.8) and the average age of controls was 65.6 years (range 40.0–97.5). The study was approved by the Human Research Ethics Committee at the Peter MacCallum Cancer Centre (Approval # 09/29) and all participating centres. All participants provided informed consent for genetic analysis of their germline DNA.

The coding region and exon–intron boundaries (10 bp of intronic sequence at each site) of 1317 genes (phase 1) and 159 genes (phase 2) (Supplementary Data) were amplified from germline DNA using custom designed HaloPlex Targeted Enrichment Assay panels (Agilent Technologies, Santa Clara, CA) as described previously^43^. Full details on sequencing alignment, variant calling and variant filters are described in the supplementary online methods.

### Statistical analysis

*P*-values were computed by Fisher’s exact test (2-sided) or Chi-squared test with Yates correction using R version 3.6.1^44^. A *p*-value of <0.05 was considered as statistically significant and Benjamini–Hochberg adjustment (BH) was used for multiple test corrections^45^. Haldane–Anscombe correction was used to calculate the odds ratio where a variant frequency of zero. The conditional Maximum Likelihood Estimate was used for the calculation of confidence intervals. Population attributable fraction (PAF) was calculated using the frequency of variants in the Lifepool control group according to Levin’s formula^46^. The relative risk estimate used in PAF calculation was based on the odds ratio observed in case-control data adjusted for population background risk of breast cancer^47^.

### Reporting summary

Further information on research design is available in the Nature Research Reporting Summary linked to this article.

## Supplementary information

Supplementary Information

Supplementary Data 1

Reporting Summary

## Data Availability

The data generated and analysed during this study are described in the following data record: 10.6084/m9.figshare.14439455^48^. The sequencing data have been deposited in the European Genotype-phenotype Archive under the following accession: https://identifiers.org/ega.dataset:EGAD00001007025 (study ID: EGAS00001005043). These data include: sequencing alignment, variant calling and variant filters, principal component analysis and identity-by-state analysis. Additionally, the following data are not openly available to protect patient privacy: FCC patient database. Data requests for these data should be made to the corresponding author.

## Code availability

Standard R codes were used. Code requests should be addressed to I.G.C.
