# Supplementary material for: Investigation of monogenic causes of familial breast cancer: data from the BEACCON case-control study
Source: NPJ Breast Cancer. 2021 Jun 11;7:76. doi: 10.1038/s41523-021-00279-9 (PMC8196173; doi:10.1038/s41523-021-00279-9)
Supplement: Supplementary file 1 — Supplementary Information [file 41523_2021_279_MOESM1_ESM.pdf]

Supplementary Information

Contains supplementary figures, tables and online methods.

Supplementary Figures

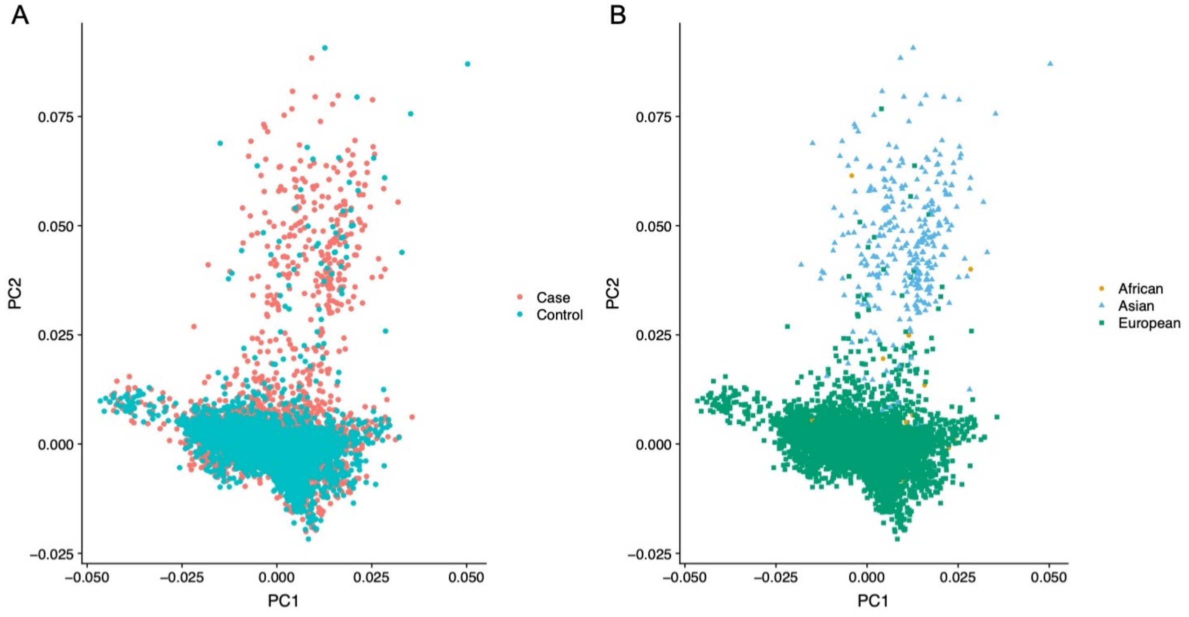

Supplementary Figure 1. Principal component analysis (PCA) of subjects in 5,770 cases and 5,741 controls using all genetic variants identified in BEACCON study. Each graph shows the first two principal components (PC1 & PC2). (A) Ancestry distribution between case subjects (red dot) and control subjects (blue dot). (B) Clusters of European (green square), Asian (blue triangle) and African ancestry (yellow dot) identified in all sequenced case and control subjects. Among 5,770 familial breast and/or ovarian cases and 5,741 controls, PCA analysis showed that both the case and control groups were predominantly of European ancestry (95.3% cases and 98.8% controls), while a small difference was seen in the minor race groups, with 0.3% African and 4.3% Asian ancestry in the cases and 0.06% African and 1.1% Asian ancestry in the controls. The difference in Asian component results in identifying a number of LoF variants contributed primarily by Asian subjects (*MSH6* p.Lys1358AspfsTer2 in 14 cases and 4 controls, East Asian (EAS) MAF 0.0324 in gnomAD; *MUTYH* c.925-2A>G in 11 cases

and 0 controls, EAS MAF 0.0155); *SLC5A4* p.Arg267Ter in 3 cases and 3 controls, EAS MAF 0.0229; and *SPTBN5* p.Gln72Ter, in 4 cases and 4 controls, EAS MAF 0.0307) that were excluded in the analysis.

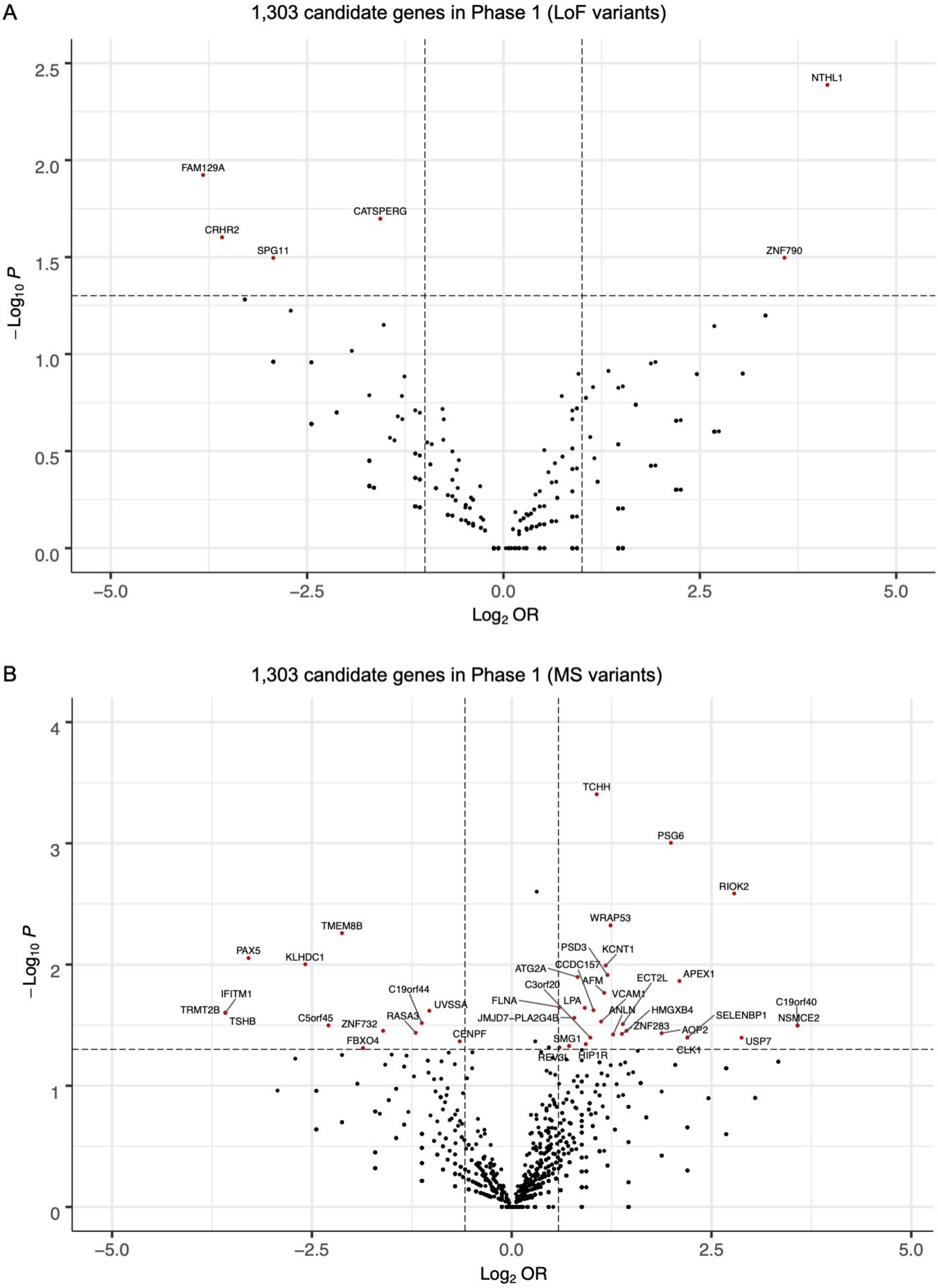
A
1,303 candidate genes in Phase 1 (LoF variants)
NTHL1
FAM129A
CATSPERG
CRHR2
SPG11
ZNF790
2.5
2.0
1.5
1.0
0.5
0.0
−5.0
−2.5
0.0
2.5
5.0
$-\text{Log}_{10}\,P$
Log_2 OR
B
1,303 candidate genes in Phase 1 (MS variants)
TCHH
PSG6
RIOK2
WRAP53
TMEM8B
PSD3
KCNT1
PAX5
KLHDC1
CCDC157
ATG2A
APEX1
AFM
ECT2L
C3orf20
C19orf44
IFITM1
VCAM1
FLNA
LPA
UVSSA
C19orf40
TRMT2B
HMGXB4
ANLN
JMJD7−PLA2G4B
SELENBP1
TSHB
C5orf45
RASA3
NSMCE2
ZNF732
ZNF283
AQP2
CENPF
SMG1
USP7
FBXO4
CLK1
REV3L
HIP1R
4
3
2
1
0
−5.0
−2.5
0.0
2.5
5.0
$-\text{Log}_{10}\,P$
Log_2 OR

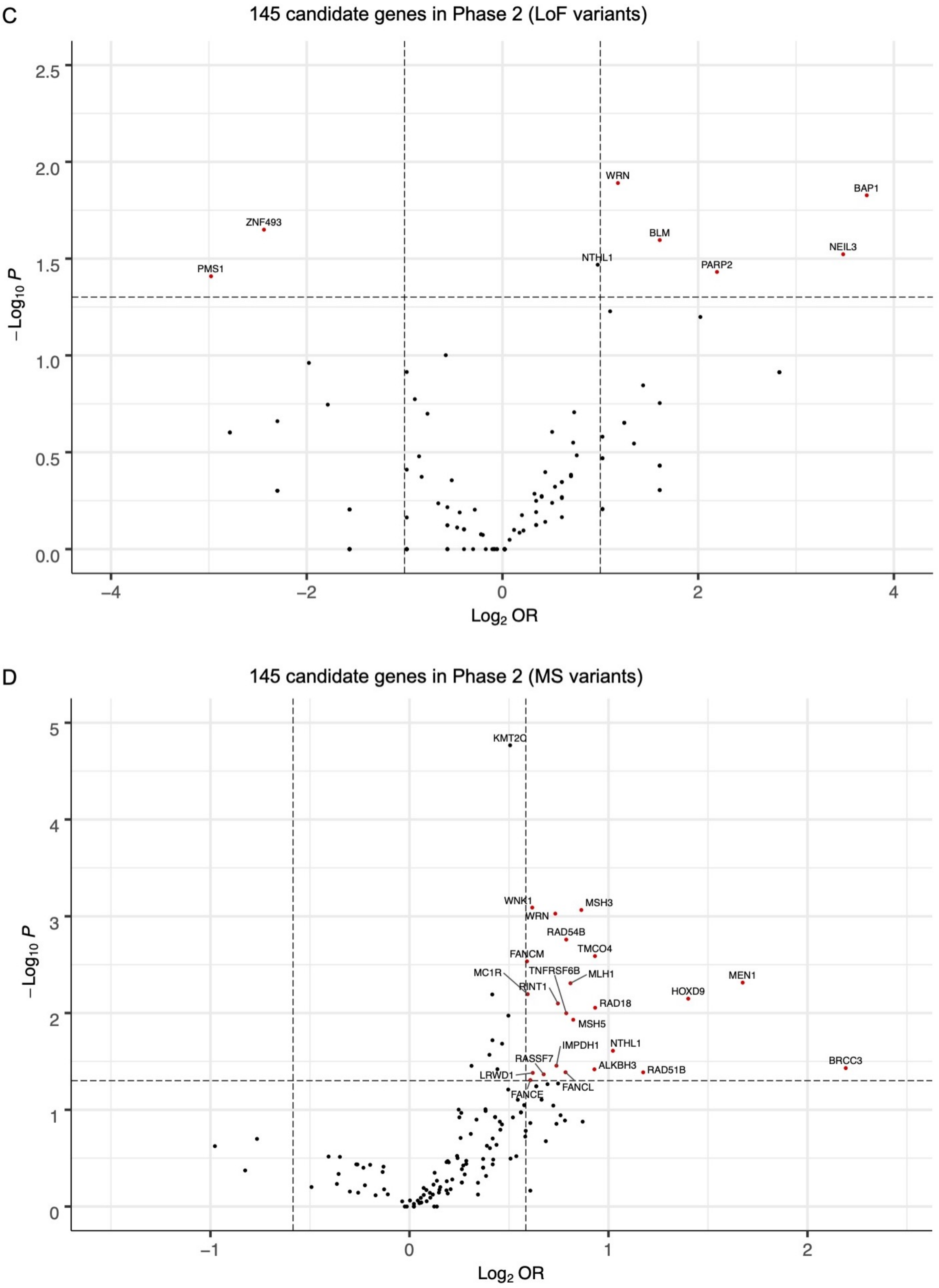

Supplementary Figure 2: Volcano Plots showing progress of candidate gene selection from Phase 1 to Phase 2. A, B, the distribution of 1,303 candidate genes sequenced in Phase 1 in up to 1,990 cases and 1902 controls by OR and p-values based on LoF or MS variants; C, D,

the distribution of 145 candidate genes sequenced in Phase 2 in up to 3,780 cases and 3,839 controls by OR and p-values based on LoF or MS variants. The horizontal axis is the log 2-fold change (log2(OR)) between case and control groups, whereas the vertical axis represents the reliability of the result (-Log10(P)). The horizontal dash line identifies the p-value threshold ($P \leq 0.05$, without multiple testing adjustment). Two vertical dash lines show the threshold of fold change (OR > 2 or < 0.50 for LoF variants and OR > 1.50 or < 0.67 for MS variants). Each spot represents a gene that was sequenced with the colour shading indicating genes that showed odds ratio and p-value above (red shading) or below (black shading) the thresholds.

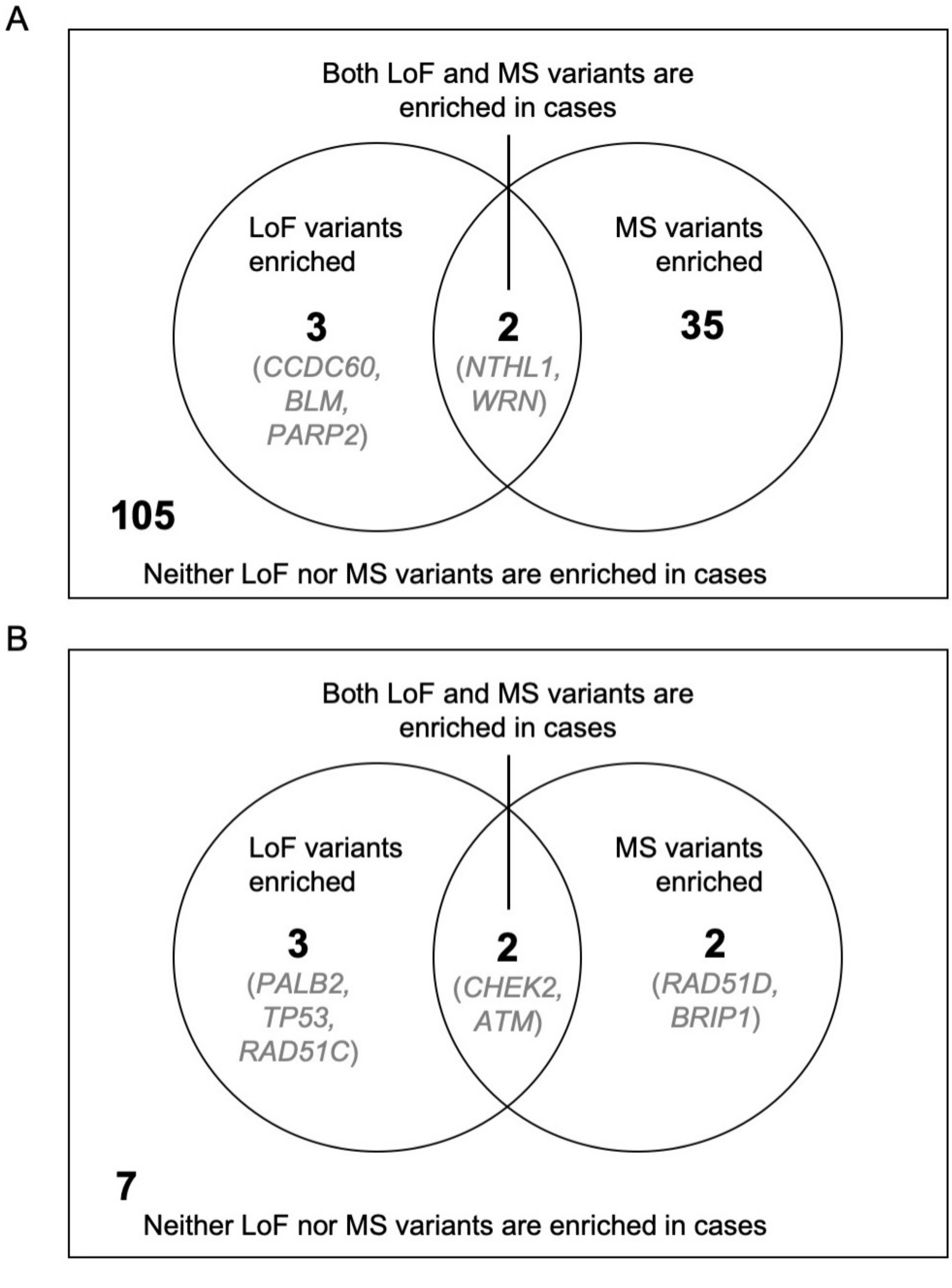
A
Both LoF and MS variants are enriched in cases
LoF variants enriched
3
(CCDC60, BLM, PARP2)
2
(NTHL1, WRN)
MS variants enriched
35
105
Neither LoF nor MS variants are enriched in cases
B
Both LoF and MS variants are enriched in cases
LoF variants enriched
3
(PALB2, TP53, RAD51C)
2
(CHEK2, ATM)
MS variants enriched
2
(RAD51D, BRIP1)
7
Neither LoF nor MS variants are enriched in cases

Supplementary Fig. 3: Venn diagram showing distribution of (A) 145 candidate genes and (B) 14 previously reported HBOC genes according to enrichment in LoF variants and/or enrichment in MS variants ($p<0.05$, $OR>1$).

Supplementary Tables

Supplementary Table 1. (A) Likely pathogenic missense variants in *ATM*, *CHEK2* and *PALB2* in cases and controls selected by rarity or deleterious *in silico* properties. MAF, minor allele frequency in gnomAD. (B) Rare (MAF < 0.001) missense variants of *PALB2* in cases compared to controls by location in different functional domains.

(A)

**Likely pathogenic missense variants**		***ATM* ***			***CHEK2* †**			***PALB2***		
		Case n=5,770	Control n=5741	OR (95%CI)	Case n=5,770	Control n=5741	OR (95%CI)	Case n=5,770	Control n=5741	OR (95%CI)
**MAF**	<0.001	265	207	1.29 (1.06-1.56)	131	71	1.86 (1.38-2.52)	118	115	1.02 (0.78-1.34)
	<0.0005	209	154	1.36 (1.10-1.70)	124	69	1.81 (1.33-2.47)	93	84	1.10 (0.81-1.50)
	<0.00005	105	64	1.64 (1.19-2.29)	48	30	1.60 (0.99-2.61)	49	46	1.06 (0.69-1.62)
**CADD**	>15	148	100	1.48 (1.14-1.94)	100	47	2.14 (1.49-3.10)	85	69	1.23 (0.88-1.72)
	>20	92	44	2.10 (1.45-3.08)	41	21	1.95 (1.12-3.48)	37	41	0.90 (0.56-1.44)
	>25	39	21	1.85 (1.06-3.32)	9	7	1.28 (0.42-4.05)	1	4	0.25 (0.01-2.51)
**REVEL**	>0.3	132	86	1.54 (1.16-2.05)	80	31	2.59 (1.69-4.06)	30	36	0.83 (0.49-1.39)
	>0.5	91	44	2.07 (1.43-3.05)	47	18	2.61 (1.49-4.78)	0	0	0
	>0.7	49	16	3.06 (1.71-5.78)	34	12	2.83 (1.43-6.01)	0	0	0

* Excluding the pathogenic variant c.7271T>G (p.Val2424Gly, NM_000051.3, rs28904921).

† The analysis did not include the low-penetrance variant in *CHEK2*, c.470T>C (p.Ile157Thr, NM_007194.4, rs17879961) which had a MAF 0.005 in GnomAD

(B)

***PALB2* functional Domain (amino acid, aa)**	**Cases**	**Controls**	**Total No. cases**	**Total No. controls**	**OR (95% CI)**	**P ***
DNA binding (1-579 aa)	39	46	5770	5741	0.84 (0.53-1.32)	0.45
Interaction with BRCA1 (1-319 aa)	29	30	5770	5741	0.96 (0.56-1.66)	0.90
Interaction with RAD51 (1-200 aa)	18	24	5770	5741	0.75 (0.38-1.43)	0.36
Oligomerization and focal concentration at DNA damage sites (1-160 aa)	18	20	5770	5741	0.90 (0.45-1.78)	0.75
Interaction with POLH and POLH DNA synthesis stimulation (775-1186 aa)	69	57	5770	5741	1.21 (0.84-1.75)	0.33
Interaction with RAD51, BRCA2 and POLH (853-1186 aa)	64	50	5770	5741	1.28 (0.87-1.89)	0.22

* P values were calculated by Fisher's exact test, 2-sided.

Supplementary Table 2. Enrichment of LoF and MS variant in the cases compare to the controls in each sequencing phases.

Sequencing Phase	No. of genes	No. of cases	No. of controls	No. of rare variants			No. of nucleotides sequenced		OR	P *
				Type	Case	Control	Case	Control		
Phase 1	1,303	Up to 1,990	Up to 1,902	LoF	2346	2044	1.80E+10	1.76E+10	1.13	7.42E-05
				MS	21192	17765	1.80E+10	1.76E+10	1.17	8.62E-55
Phase 2	145	3,780	3,839	LoF	1064	1006	8.45E+09	8.70E+09	1.09	0.05
				MS	10689	8752	8.45E+09	8.70E+09	1.26	3.83E-57
Combined †	145	Up to 5,770	Up to 5,741	LoF	1330	1073	1.06E+10	1.08E+10	1.27	9.05E-09
				MS	12708	10206	1.06E+10	1.08E+10	1.27	3.96E-73

* P values were calculated by Chi-squared test with Yates correction.

† Combined analysis included data for the candidate genes (n=145) sequenced in both Phase 1 and Phase 2.

Supplementary Table 3. (A) List of genes in DNA repair pathways, (B) LoF and MS variant carrier frequency in cases and controls of genes in DNA repair pathways

(A)

**Functional Pathway**	**Genes**
Base excision repair	*ALKBH1, ALKBH2, ALKBH3, APEX1, APEX2, MPG, MUTYH, NEIL1, NEIL2, NEIL3, NTHL1, OGG1, PARP1, PARP4, SMUG1, UNG, XRCC1*
Homologous recombination repair	*BAP1, BLM, ERCC4, FAN1, FANCA, FANCB, FANCC, FANCD2, FANCE, FANCF, FANCG, FANCI, FANCL, FANCM, LIG4, SLX4, WRN*
Mismatch repair	*MLH1*, MLH3, MSH2*, MSH3, MSH4, MSH5, MSH6*, PMS1, PMS2**

genes marks with asterisks (*) are Lynch syndrome genes

(B)

**Pathway**	**LoF variants**				**MS variants**			
	**Case (%) N=4,807**	**Control (%) N=4,782**	**OR (95%CI)**	**P**	**Case (%) N=4,807**	**Control (%) N=4,782**	**OR (95%CI)**	**P**
Homologous recombination repair	180 (3.74%)	121 (2.53%)	1.48 (1.18-1.91)	0.001	1360 (28.29%)	1169 (24.45%)	1.16 (1.11-1.34)	0.00002
Base excision repair	161 (3.35%)	121 (2.53%)	1.32 (1.04-1.71)	0.02	715 (14.87%)	607 (12.69%)	1.17 (1.07-1.35)	0.002
Lynch syndrome genes	32 (0.67%)	34 (0.71%)	0.94 (0.56-1.57)	0.81	344 (7.16%)	295 (6.17%)	1.16 (0.99-1.38)	0.05
All mismatch repair genes	64 (1.33%)	65 (1.36%)	0.98 (0.68-1.41)	1.00	707 (14.71%)	574 (12.00%)	1.23 (1.12-1.43)	0.0001

Supplementary Table 4. Tumour and family characteristics observed in the Variant in Practice (ViP) study samples (n = 3,065).

	Number	%
**BC index patients, overall**	3065	
Bilateral BC	354	11.5%
BC and OC affected	131	4.3%
**BC Family history**		
≥ one 1st degree relatives affected with BC	1327	43.3%
≥ two 1st degree relatives affected with BC	285	9.3%
At least one age ≤ 40 years	186	6.1%
≥ two 2nd degree relatives affected with BC	443	14.5%
**OC Family History**		
1st degree relatives affected with OC	229	7.5%
2nd degree relatives affected with OC	223	7.5%
**BC index patients Histopathology Type (n=2,710)**		
Ductal	2377	87.7%
Ductal, medullary	58	2.1%
Lobular	193	7.1%
Mixed Ductal and Lobular	78	2.9%
Adenocarcinoma	3	0.11%
Sarcoma	1	0.04%
Unknown	355	
**BC index patients, age at first BC diagnosis**		
<30	166	5.4%
30-39	824	26.9%
40-49	1060	34.6%
50-59	636	20.8%
≥60	376	12.3%
Unknown	3	
**BC index patients, Hormone receptor status at first BC diagnosis**		
ER+/PR+	1429	46.7%
HER2+	224	15.6%
HER2-	889	62.2%
HER2 unknown	316	22.1%
ER+/PR-	226	7.4%
HER2+	56	24.8%

		HER2-	137	60.6%
		HER2 unknown	33	14.6%
	ER-/PR+		64	2.1%
		HER2+	14	21.9%
		HER2-	32	50.0%
		HER2 unknown	18	28.1%
	ER-/PR-		798	26.0%
		HER2+	159	19.9%
		HER2-	550	68.9%
		HER2 unknown	89	11.2%
	ER/PR/HER2 Unknown		548	17.9%
**OC Histopathology Type (n=92)**				
	High grade serous		36	39.1%
	Low grade serous		2	2.2%
	Serous (unspecified)		3	3.1%
	Endometroid		23	25.0%
	Clear cell		9	9.8%
	Mucinous		4	4.3%
	Others		15	16.3%
	Unknown		39	

Supplementary table 5. Analysis of multiple LoF variants carriers. (A) Frequency of multiple LoF variants carriers in case and control cohorts. (B) Observed and expected frequency of multiple LoF variants carriers in case and control cohorts.

(A)

No. of LoF variants	No. of cases		No. of controls		OR (95%CI)	P *
	LoF carrier	Total	LoF carrier	Total		
0	3747	4807	3837	4782	0.87 (0.79-0.96)	0.006
1	1041	4807	834	4782	1.31 (1.18-1.45)	1.95E-07
2	169	4807	105	4782	1.62 (1.26-2.1)	1.07E-04
3	19	4807	6	4782	3.16 (1.21-9.67)	0.02

* Fisher's exact text, 2-sided.

(B)

No. of LoF variants	No. of cases N=4807			No. of controls N= 4782		
	Observed	Expected*	X^2 P †	Observed	Expected*	X^2 P †
1	1041	1066	0.55	834	851	0.67
2	169	159	0.61	105	94	0.47
3	19	16	0.73	6	7	1.00
≥ 2	188	175	0.52	111	101	0.53

* Expected value from a binomial distribution based on the overall frequency of LoF variants in cases and controls given the number of genes tested and overall coverage (>10x) of 92.0% in cases and 92.8% in controls.

† P values were calculated by chi-square test with Yates correction.

Supplementary methods

Gene panel design

This study involved two phases of sequencing (Figure 1). The first phase sequenced 14 previously reported hereditary breast and ovarian cancer (HBOC) genes and 1,303 candidate genes in a maximum of 1,990 non-BRCA1/2 cases and 1,902 population controls. The candidate genes were genes that had at least one LoF variant detected in whole-exome sequencing data from 150 breast cancer (BC) affected cases from 69 non-BRCA1/2 families [1, 2] and combined with a list of 417 genes (additional 315 genes) that had a literature-supported role in DNA repair function. The 145 candidate genes in Phase 2 consisted of the top candidate genes from phase 1 selected based on the most significant associations, combined with a list of 41 genes involved in four DNA repair pathways (HRR, MMR, BER and DRR; 26 additional genes) according to research interest in the literature that showed enriched LoF variants in the cases compared to the controls in Phase 1 data. Together with the 14 HBOC genes, the 145 candidate genes in Phase 2 were sequenced in additional 3,780 non-BRCA1/2 cases and 3,839 controls (Supplementary Fig. 2). In addition to the HBOC and candidate genes, a total of 70 low penetrance BC associated SNPs were included in Phase 1 and Phase 2 design to calculate a polygenic risk score (PRS) described by Mavaddat *et al.* [3]. A set of 74 common SNPs that were verified by previous studies to exhibit substantially different frequencies between different populations (Ancestry Informative Markers, AIMs) [4-6] was genotyped in 3409 subjects (1747 cases and 1662 controls) in Phase 2 to provide ethnicity background information in principle component analysis. A complete list of genes and respective sample size of each phase are included in Supplementary Table 3.

Massively parallel sequencing

The coding region and exon-intron boundaries (10 bp of each intron from both sides) of 1,317 genes (phase 1) and 159 genes (phase 2) (Supplementary list 1) were amplified from germline DNA using custom designed HaloPlex Targeted Enrichment Assay panels (Agilent

Technologies, Santa Clara, CA). The libraries were sequenced on a HiSeq2500 Genome Analyzer (Illumina, San Diego, CA) as described previously [7-10]. Samples that did not reach a minimum of 80% of bases covered at 10x coverage were excluded from further analysis.

Sequencing alignment, variant calling and variant filters

Paired-end sequencing alignment was performed using the Burrows-Wheeler Alignment tool to the g1 k x27 h19 reference genome [11]. Indel realignment and base quality score recalibration were performed using the Genome Analysis Toolkit (GATK) [12]. Indel and SNP variant calling was carried out using GATK Haplotype caller, UnifiedGenotyper v2.4 [13] and Platypus [14]. Annotation of variants was performed using the Ensembl Variant Effect Predictor [15]. Loss-of-function (LoF) variants were defined as stop gained, frame-shift or essential splice site variants. LoF and missense (MS) variants were identified relative to the CANONICAL transcript of individual gene according to Ensemble database, and had passed various quality filters including: passing at least two the three variant callers, alternative allele proportion ≥20% individually or ≥35% for recurrent variants. A minor allele frequency (MAF) ≤0.005 in non-Finnish European and overall cohorts in gnomAD (Version 2.1, released 17 October 2018) was used for LoF variants and ≤0.001 for MS variants [16]. Pathogenic variants in HBOC genes were defined as LoF variants and known pathogenic MS variants reported in the ClinVar database. *In silico* assessment tools Condel[17], PolyPhen2[18], SIFT[19], CADD[20] and REVEL[21] were used to predict the likely pathogenicity of missense variants. Manual examination of BAM files using Integrative Genomics Viewer (IGV) [22] and Sanger sequencing was carried out for top candidate genes to screen for sequencing artefacts. The top candidate gene list was manually curated to remove genes that were unlikely to be high risk BC genes with high frequency of variants (>15% in case and/or control cohort).

Principal component analysis

Principal Component Analysis (PCA) was applied to variants from the sequencing data, as described previously [10]. Sequencing data of the 74 Ancestry Informative Markers (AIMs) [4-6] in 3409 subjects (1747 cases and 1662 controls, in Phase 2) were used to calibrate the ancestry clustering in PCA for the whole cohort using all genetic variants in the entire targeted regions in all sequencing phases. A total of 5,770 familial breast cancer cases and 5,741 controls was analysed by PCA to determine their ethnicity background.

Identity-by-state analysis

Identity-By-State (IBS) analysis of raw SNP data was performed using PLINK (v1.9) [23]. Sample pairs with significantly high IBS scores were flagged as potential duplicate or related samples. Flagged samples were forwarded to clinical collaborators for validation, resulting in 56 total confirmed replicates or likely duplicates (based on identical initials and date of birth) and 6 possibly mislabelled samples that have been excluded from study.

Phenotypic subgroup analysis

Data on tumour pathology and family history was obtained from the ViP Study (n=3,065) and collated with sequencing results. Cohort characteristics are presented in Results and supplementary Table 1. Fisher's exact test was used to examine sub-cohort case-control associations between germline pathogenic variants across all 159 genes from Phase 2 with tumor pathology and family history phenotypes through a contingency table of cohort distribution and gene variant carrier distribution. An odds ratio cut-off of 1 was applied to select for positive associations. Examined phenotypes include ER status, PR status, HER2 status, triple-negative subtype, lobular subtype, primary ovarian cancer and first-degree ovarian cancer. In cases where information was not available, the subjects were excluded from the analysis.

Statistical analysis

Statistical analyses were performed using R version 3.6.1 (R Foundation for Statistical Computing, Vienna, Austria). The significance of results was assessed using Fisher test p-value via R package Psych. Volcano and Forest plots were constructed using R package EnhancedVolcano and Forestplot respectively. Overall enrichment of variants in case cohort vs control cohort was calculated based on frequency of variants among total targeted sequencing region (≥10-fold reads), approximating one nucleotide is affected by each variant, and accounting for both alleles, variation between samples and across panels. As a reference to BEACCON control cohort, frequency of gene variants in gnomAD database was determined as number of variants detected (filtered high impact LoF variants and filtered MS variants) against maximum number of alleles screened, noting that the frequency of variants in BEACCON control is presented as per individual. PRS was calculated based on 70 low penetrance BC associated SNPs following a multiplicative risk model (calculated by sum of the minor alleles weighted by the per-allele log OR) described by Mavaddat *et al*. [3].

Reference

1. Complexo, Southey MC, Park DJ*, et al.* COMPLEXO: identifying the missing heritability of breast cancer via next generation collaboration. Breast cancer research : BCR 2013;15(3):402-402.

2. Thompson ER, Doyle MA, Ryland GL*, et al.* Exome Sequencing Identifies Rare Deleterious Mutations in DNA Repair Genes FANCC and BLM as Potential Breast Cancer Susceptibility Alleles. PLOS Genetics 2012;8(9):e1002894.

3. Mavaddat N, Pharoah PD, Michailidou K*, et al.* Prediction of breast cancer risk based on profiling with common genetic variants. J Natl Cancer Inst 2015;107(5).

4. Kidd JR, Friedlaender FR, Speed WC*, et al.* Analyses of a set of 128 ancestry informative single-nucleotide polymorphisms in a global set of 119 population samples. Investig Genet 2011;2(1):1.

5. Kosoy R, Nassir R, Tian C*, et al.* Ancestry informative marker sets for determining continental origin and admixture proportions in common populations in America. Hum Mutat 2009;30(1):69-78.

6. Nassir R, Kosoy R, Tian C*, et al.* An ancestry informative marker set for determining continental origin: validation and extension using human genome diversity panels. BMC Genet 2009;10:39.

7. Li N, Rowley SM, Thompson ER*, et al.* Evaluating the breast cancer predisposition role of rare variants in genes associated with low-penetrance breast cancer risk SNPs. Breast Cancer Res 2018;20(1):3.

8. Li N, Rowley SM, Goode DL*, et al.* Mutations in RECQL are not associated with breast cancer risk in an Australian population. Nat Genet 2018;50(10):1346-1348.

9. Forbes SA, Beare D, Bindal N*, et al.* COSMIC: High-Resolution Cancer Genetics Using the Catalogue of Somatic Mutations in Cancer. Curr Protoc Hum Genet 2016;91:10.11.1-10.11.37.

10. Li N, Thompson ER, Rowley SM*, et al.* Reevaluation of RINT1 as a breast cancer predisposition gene. Breast Cancer Res Treat 2016;159(2):385-92.

11. Li H. Aligning sequence reads, clone sequences and assembly contigs with BWA-MEM. arXiv preprint arXiv:1303.3997 2013.

12. Stormo GD. An Overview of RNA Sequence Analyses: Structure Prediction, ncRNA Gene Identification, and RNAi Design. Curr Protoc Bioinformatics 2013;43:12.1.1-3.

13. Van der Auwera GA, Carneiro MO, Hartl C*, et al.* From FastQ data to high confidence variant calls: the Genome Analysis Toolkit best practices pipeline. Curr Protoc Bioinformatics 2013;43:11 10 1-11 10 33.

14. Rimmer A, Phan H, Mathieson I*, et al.* Integrating mapping-, assembly- and haplotype-based approaches for calling variants in clinical sequencing applications. Nat Genet 2014;46(8):912-918.

15. McLaren W, Gil L, Hunt SE*, et al.* The Ensembl Variant Effect Predictor. Genome Biol 2016;17(1):122.

16. Lek M, Karczewski KJ, Minikel EV*, et al.* Analysis of protein-coding genetic variation in 60,706 humans. Nature 2016;536(7616):285-91.

17. Gonzalez-Perez A, Lopez-Bigas N. Improving the assessment of the outcome of nonsynonymous SNVs with a consensus deleteriousness score, Condel. Am J Hum Genet 2011;88(4):440-9.

18. Adzhubei IA, Schmidt S, Peshkin L*, et al.* A method and server for predicting damaging missense mutations. Nat Methods 2010;7(4):248-9.

19. Ng PC, Henikoff S. Predicting deleterious amino acid substitutions. Genome Res 2001;11(5):863-74.

20. Kircher M, Witten DM, Jain P*, et al.* A general framework for estimating the relative pathogenicity of human genetic variants. Nat Genet 2014;46(3):310-5.

21. Ioannidis NM, Rothstein JH, Pejaver V*, et al.* REVEL: An Ensemble Method for Predicting the Pathogenicity of Rare Missense Variants. Am J Hum Genet 2016;99(4):877-885.

22. Thorvaldsdottir H, Robinson JT, Mesirov JP. Integrative Genomics Viewer (IGV): high-performance genomics data visualization and exploration. Brief Bioinform 2013;14(2):178-92.
23. Chang CC, Chow CC, Tellier LCAM*, et al.* Second-generation PLINK: rising to the challenge of larger and richer datasets. GigaScience 2015;4(1).
